# Gait-CNN-ViT: Multi-Model Gait Recognition with Convolutional Neural Networks and Vision Transformer

**DOI:** 10.3390/s23083809

**Published:** 2023-04-07

**Authors:** Jashila Nair Mogan, Chin Poo Lee, Kian Ming Lim, Mohammed Ali, Ali Alqahtani

**Affiliations:** 1 Faculty of Information Science and Technology, Multimedia University, Melaka 75450, Malaysia; 2 Department of Computer Science, King Khalid University, Abha 61421, Saudi Arabia; 3 Center for Artificial Intelligence (CAI), King Khalid University, Abha 61421, Saudi Arabia

**Keywords:** deep learning, ensemble, gait, gait recognition

## Abstract

Gait recognition, the task of identifying an individual based on their unique walking style, can be difficult because walking styles can be influenced by external factors such as clothing, viewing angle, and carrying conditions. To address these challenges, this paper proposes a multi-model gait recognition system that integrates Convolutional Neural Networks (CNNs) and Vision Transformer. The first step in the process is to obtain a gait energy image, which is achieved by applying an averaging technique to a gait cycle. The gait energy image is then fed into three different models, DenseNet-201, VGG-16, and a Vision Transformer. These models are pre-trained and fine-tuned to encode the salient gait features that are specific to an individual’s walking style. Each model provides prediction scores for the classes based on the encoded features, and these scores are then summed and averaged to produce the final class label. The performance of this multi-model gait recognition system was evaluated on three datasets, CASIA-B, OU-ISIR dataset D, and OU-ISIR Large Population dataset. The experimental results showed substantial improvement compared to existing methods on all three datasets. The integration of CNNs and ViT allows the system to learn both the pre-defined and distinct features, providing a robust solution for gait recognition even under the influence of covariates.

## 1. Introduction

Gait recognition is a biometric technology that uses an individual’s walking pattern as a means of identification. The uniqueness of an individual’s gait comes from various traits such as stride length, arm swing, limb movement, and others, which make it difficult to conceal or imitate. Furthermore, gait recognition does not require any active participation from the subject, making it a convenient method of identification. However, the performance of gait recognition can be impacted by a number of covariates such as walking speed, viewing angle, clothing, and carrying condition. These covariates can alter the appearance of the gait pattern, causing difficulty in accurately identifying individuals.

In the past, gait recognition primarily relied on handcrafted approaches, which can be divided into two categories: model-based and model-free. Model-based approaches use a skeleton model to extract gait features, but these methods can be computationally expensive. On the other hand, model-free approaches encode gait information directly from gait silhouettes, making them more computationally efficient. Despite the promising results from these handcrafted approaches, they have limitations as they only learn pre-defined features and may neglect important information.

In recent years, deep learning approaches such as Convolutional Neural Networks (CNNs) have been widely used for recognition and classification tasks. This is due to the ability of CNNs to learn powerful and discriminative features from input images. The integration of pre-trained CNNs, such as ResNet [1], AlexNet [2], DenseNet [3], and VGG [4], is becoming popular among researchers as these models are learned from large datasets and are capable of extracting important features.

Moreover, the implementation of self-attention mechanisms in deep learning has also garnered attention due to their impressive performance in natural language processing applications, such as machine translation, next sentence prediction, and language modeling. The self-attention mechanism allows inputs to interact with each other and determines the correlation between inputs to form a representation of the sequence. Among the self-attention architectures, Transformers are particularly popular due to their versatility. While some studies have applied pre-trained models and Transformers separately, only a few have combined both approaches in their work.

This work presents a multi-model gait recognition method that leverages the strengths of DenseNet-201, VGG-16, and Vision Transformer (ViT). The choice of models is based on a variety of considerations. DenseNet-201 reduces the computation time and reduces the number of parameters by utilizing dense connectivity, which allows each layer in the network to directly connect to the features in the previous layers. Similarly, VGG-16 requires fewer parameters and is straightforward to implement. ViT, on the other hand, benefits from its multi-head attention mechanism, which extracts features from all parts of the image, thereby allowing the model to learn both local and global dependencies. The process begins by creating a Gait Energy Image (GEI) from the silhouettes by windowing them over a gait cycle. Each model then individually processes the GEIs and learns the relevant gait features. The final prediction is made by averaging the class scores generated by each model. By incorporating ensemble methods, the gait recognition system is capable of producing more accurate and reliable results, making it a valuable asset in high-stakes environments such as security systems and video surveillance. Additionally, the system’s ability to accurately identify individuals through their gait patterns can also be useful in healthcare applications, such as detecting abnormalities in walking patterns that could indicate underlying medical conditions.

The main contributions of this work can be summarized as follows:

- A multi-model approach for gait recognition, which combines the use of DenseNet-201, VGG-16, and Vision Transformer. The CNN models and Vision Transformer are utilized to extract important gait features, and a multi-layer perceptron to identify the relationship between these features and the class labels;
- An averaging ensemble technique to merge the predictions from all the models, resulting in improved performance in vision-based gait recognition.

## 2. Related Works

In the existing literature on gait recognition, researchers have proposed various approaches that can be broadly categorized into two groups: handcrafted methods and deep learning methods.

Handcrafted methods involve manual extraction of pre-defined features from gait data. This process typically involves selecting certain characteristics of the gait such as stride length, cadence, and gait rhythm, and using these features to distinguish between individuals. The drawback of handcrafted methods is that they require a significant amount of expert knowledge to identify the appropriate features and may not capture all of the nuances and complexities of gait patterns.

On the other hand, deep learning methods encode intricate features automatically, without any human intervention. These methods use neural networks to learn the underlying patterns in gait data and identify individuals based on these patterns. Deep learning methods have the advantage of being able to capture more subtle and complex features of gait patterns, leading to improved recognition accuracy compared to handcrafted methods.

### 2.1. Handcrafted Approach

Handcrafted methods in gait recognition can be divided into two categories: model-based and model-free. The model-based approach uses a human model consisting of stick figures and joints to extract motion information [5,6,7,8]. In the work of Deng et al. (2018) [9], a deterministic learning algorithm was used to encode both spatial-temporal features and kinematic features. The authors chose the width of lower limbs and the integrated silhouette region as spatial-temporal features and four lower limb joint angles as kinematic features.

Sah and Panday (2020) [10] proposed a posture-based gait recognition method using a weighted KNN algorithm. They extracted 20 coordinates from a Kinect skeletal model, which were then converted into Centre of Body (CoB) coordinates. These coordinates capture the position and dimension of various body parts in each frame.

In the work of Sharif et al. (2020) [11], three types of features were extracted: shape, geometric, and texture. The Histograms of Oriented Gradients (HOG) were used to capture shape features, while Principal Component Analysis (PCA) was applied on the HOG features to reduce their dimension. Six geometric features were calculated and five statistical measures were extracted for each feature. The texture features were learned using a Volume Local Binary Pattern (VLBP) method. Finally, the three types of features were combined using a parallel fusion method.

In contrast to the model-based approach, the model-free approach in gait recognition focuses on analyzing the motion information directly, without relying on a human model [12,13,14,15,16,17,18]. Lee et al. (2015) [19] proposed a method that creates texture descriptors of motion features over the temporal axis, called Transient Binary Patterns (TBP). The motion patterns are extracted at different scales, including pixel-wise, regional, and global. The TBPs are calculated and histogram representations are generated at each scale. Khan et al. (2019) [20] proposed a method that extracts gait features without performing the silhouette extraction process by directly obtaining dense trajectories from the gait video using optical flow. Based on the obtained trajectories, features such as HOG, histogram of optical flow (HOF), and horizontal and vertical components of optical flow are computed to capture both motion and appearance information. Mogan et al. (2020) [21] aimed to reduce the impact of noise, occlusion, and incomplete silhouettes. A set of pre-learned filters were used to convolve with the gait sequence frames, producing a set of feature maps for each frame. The gradients of each feature map were then calculated, and subjects were classified using the Euclidean distance and majority voting.

### 2.2. Deep Learning

In recent years, deep learning networks have become popular in processing unstructured data such as texts and images. These networks are designed to automatically extract prominent information from the data, minimizing human involvement. One of the most commonly used deep learning networks for image recognition and classification is Convolutional Neural Network (CNN).

Wu et al. (2018) [22] proposed a CNN model that consisted of two 2D convolutional layers, two max-pooling layers, and a fully connected layer. The max-pooling layers reduced the impact of deformations and noise, while the convolutional layers extracted the features. Logistic loss function was used to determine the class label.

Wang et al. (2020) [23] presented a multichannel CNN, which accepted three input images. The model consisted of five convolution layers, five pooling layers, one fusion layer, one fully connected layer, and a classifier layer. The features were extracted simultaneously from the three channels, which consisted of different convolutional kernels, and the differences between the two channels were computed in the fusion layer. Softmax function was applied in the classification stage.

Gul et al. (2021) [24] explored the use of 3D CNN to learn spatiotemporal gait features. The network consisted of four convolution layers, two pooling layers, two fully connected layers, and a classifier layer. Batch normalization technique was applied in the last convolution layer.

Han et al. (2022) [25] studied the use of angular Softmax loss to learn diverse features and triplet loss to produce more distinctive features. The study was conducted using GaitSet [26] as the backbone network and a batch normalization layer was added after the feature extraction layers to improve the loss functions.

Recently, researchers have started to apply the transfer learning technique on gait recognition by utilizing pre-trained networks. In 2017, Li et al. [27] used Joint Bayesian to calculate view variance and performed classification based on the highest similarity score using VGG-D model. In 2019, Arshad et al. [28] utilized VGG-19 and AlexNet models to learn the gait features and combined the acquired features using a parallel fusion approach. The optimal set of features was determined by calculating the entropy and skewness of the fused features. In 2022, Mehmood et al. [29] selected the best extracted features using Kurtosis and Principle Score techniques, and employed a pre-trained VGG-16 model to extract significant features. The One against All Multi Support Vector Machine (OAMSVM) was used for classification.

In the past few years, researchers have turned to ensemble methods to improve the accuracy of gait recognition compared to using a single model. The aim of using ensemble methods is to reduce noise and increase stability. For instance, Ghaeminia et al. (2019) [30] developed a method that converts gait energy into a template, using spatial and temporal filtering to extract the spatio-temporal features. During the classification stage, a classifier ensemble was employed, consisting of random subspace sampling, to improve recognition and reduce dimensionality. Wang and Yan (2021) [31] also employed ensemble learning in their gait recognition system, utilizing a primary classifier and a secondary classifier. The primary classifier consisted of five CNN models with various kernel and padding sizes, while the secondary classifier was used to ensemble the results of the primary classifier.

Other than that, there has been growing interest in incorporating transformers in CNN architectures for gait recognition. The self-attention mechanism of transformers allows it to track the association among the input sequence. For instance, Li et al. (2019) [32] proposed a CNN-based network that consists of joint intensity metric estimation net, joint intensity transformer, and discrimination net. The network evaluates joint intensity and spatial metrics to reduce the impact of clothing and carrying conditions. Similarly, Xu et al. (2020) [33] introduced a pairwise spatial transformer network to address intra-subject and inter-subject variations in gait recognition. The network uses the pairwise spatial transformer to convert pairs of different view inputs into an intermediate view, and then feeds it into a recognition network to identify the disparity between the pair of inputs. Wang and Yan (2021) [34] developed a network that takes in two GEIs as inputs and encodes both non-local and regionalized features using a self-attention mechanism during the classification stage. Pinčić et al. (2022) [35] utilized the DINO model with Vision Transformer architecture to pre-train the feature extractor to learn the gait features. The extracted features were then fed into a fully connected neural network to classify the subjects. Mogan et al. (2022) [36] proposed a method called Gait-ViT that utilized a pre-trained Vision Transformer (ViT) model for gait feature learning. In Gait-ViT, the ViT model was fine-tuned on gait recognition datasets to learn discriminative gait features. To predict the identity of the individual, a multi-layer perceptron (MLP) was employed. The MLP took the extracted features from the ViT model as input and predicted the identity of the individual.

The use of pre-trained models and transformer models have traditionally been utilized separately. However, this work presents an ensemble method that combines the best of both worlds, using a pre-trained DenseNet-201 model, a pre-trained VGG-16 model, and a pre-trained Vision Transformer model to obtain optimal performance in gait recognition.

## 3. Gait Recognition

**Figure 1:**
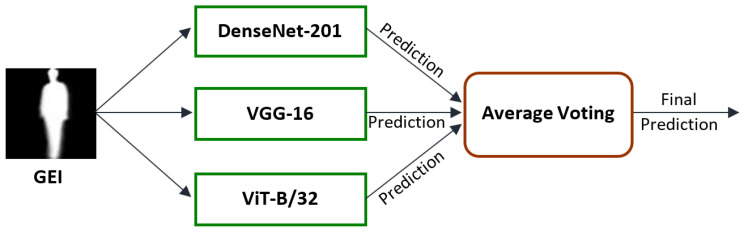
Architecture of the ensemble method.

The gait recognition process starts by obtaining Gait Energy Images (GEIs) through windowing the gait images over a gait cycle. Then, each of the three models is used to learn deep gait features from the GEIs. Each model produces an output score, which is then combined and averaged to produce the final prediction. The architecture of the proposed ensemble method is shown in Figure 1. This combination of models not only reduces the computational resources required, but also enables the extraction of low and high level features for improved gait recognition.

### 3.1. Gait Energy Image

**Figure 2:**
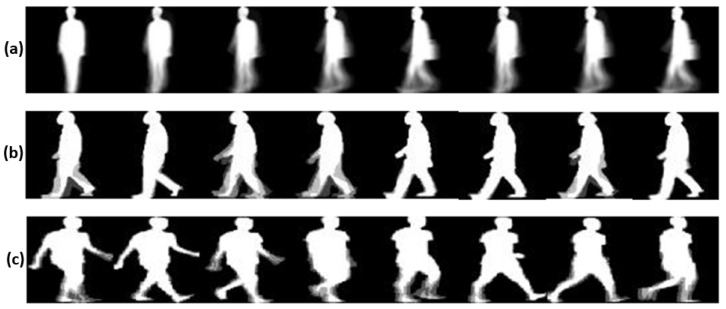
Sample GEIs from (**a**) CASIA-B dataset, (**b**) OU-ISIR D dataset, and (**c**) OU-LP dataset.

Gait Energy Image (GEI) [37] is a spatio-temporal representation, which is obtained by averaging the frames over a gait cycle. The averaging process helps to suppress the noise in the silhouettes, making GEIs robust to noise and incomplete silhouettes. GEIs are achromatic images and therefore require low computational resources. The GEI, *G*, is calculated as follows:

(1) $$G=\frac{1}{N}\sum_{t=1}^{N}I_{t}\left(x,y\right)$$

where *N* is the total number of frames of a gait cycle and $I_{t}\left(x,y\right)$ is the gait silhouette at pixel coordinate (x,y) at time *t*. Some sample GEIs of three different datasets are shown in Figure 2.

### 3.2. Network Architecture

The network architecture of the proposed ensemble method is designed to maximize the accuracy of gait recognition. The method incorporates three models, DenseNet-201, VGG-16, and Vision Transformer, each of which are used to extract deep gait features from the Gait Energy Images (GEIs). The models are trained to learn the important gait features and establish a relationship between the extracted features and the corresponding class labels. All of the models are fine-tuned on the CASIA-B dataset to optimize their hyperparameters for the gait recognition task. The combination of these three models provides a comprehensive representation of gait features that helps to improve the recognition accuracy.

#### 3.2.1. DenseNet-201 with Multilayer Perceptron

In transfer learning, a pre-trained model, which has already been trained on a large dataset for a specific task, is utilized for another downstream task. The goal of transfer learning is to reduce the amount of computational resources required for training a model from scratch and to improve the generalization of the model for the new task. The transfer learning process involves either unfreezing a part or the entire pre-trained model and fine-tuning it to suit the new task. The fine-tuning process involves adding additional dense layers and a classifier layer to the pre-trained model, depending on the complexity of the new task. This technique allows the pre-trained model to adapt to the new task and produce better results than training a model from scratch.

**Figure 3:**
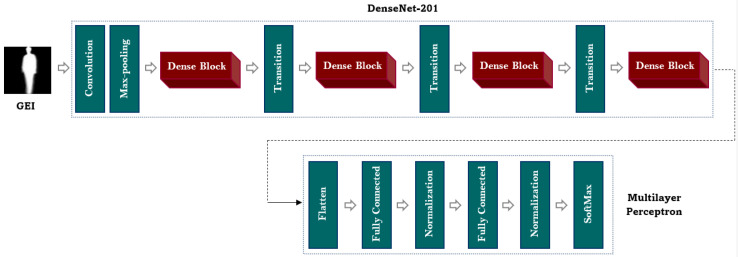
Architecture of the DenseNet-201 with the Multilayer Perceptron model.

In this study, the pre-trained DenseNet-201 model with Multilayer Perceptron is utilized for the gait recognition task. The architecture of the DenseNet-201 model with Multilayer Perceptron is shown in Figure 3.

- Densenet-201: The DenseNet-201 model was originally trained on the ImageNet dataset and consists of a convolution layer, a max-pooling layer, four dense blocks, and three transition layers. The preceding layers are directly connected to the succeeding layers in the model, which allows the feature maps of the prior layer to be concatenated with the latter layers. This intensifies the flow of information between the layers and enables the model to effectively capture and extract the crucial gait features. The concatenation of the features of the layers is described below:
  (2) $$f_{l}=H_{l}\left(\left[f_{0},f_{1},\ldots ,f_{l-1}\right]\right)$$
  where *l* is the layer and $\left[f_{0},f_{1},...,f_{l-1}\right]$ is the feature concatenation. $H_{l}\left(.\right)$ is a composite function that involves three subsequent operations such as batch normalization, ReLU activation, and a 3 × 3 convolution operation. In order to modify the size of feature maps, dense blocks are added into the model. Each dense block contains a bottleneck layer with 1 × 1 convolution, followed by a 3 × 3 convolution, both preceded by batch normalization and ReLU activation (BN-ReLU-Conv(1 × 1)-BN-ReLU-Conv(3 × 3)). The purpose of the bottleneck layer is to reduce the number of input features, making the network more computationally efficient. Transition layers are inserted after every dense block except the last one to reduce the original dimension of the feature maps by half. The transition layers perform 1×1 convolution followed by 2 × 2 average pooling. The impact of each layer in adding new information to the network’s collective knowledge is determined by using a small growth rate.
- Multilayer Perceptron: The features produced by the DenseNet-201 are passed through a multilayer perceptron (MLP) for further processing. The MLP consists of 2 fully connected layers with 512 neurons, 2 batch normalization layers, and a classifier layer. The fully connected layers are used to establish a relationship between the input features and the target classes. Each of the fully connected layers is followed by a batch normalization layer, which helps normalize the outputs of the fully connected layers in mini-batches to speed up the training process. The activation function used in the fully connected layers is the leaky rectified linear unit (Leaky ReLU), defined as:
  (3) $$f\left(x\right)=\left\{\begin{array}{cc} x & x>0\\ \alpha x & x⩽0 \end{array}\right\}$$
  where α is a constant that controls the slope of the function for negative values of *x*, thus preserving both positive and negative outputs from the neurons.
  To prevent overfitting, a dropout layer is applied after every fully connected layer. In the dropout layer, a certain percentage of neurons are randomly dropped during each iteration of the training process. This helps in reducing the dependence of the network on a particular neuron, thereby increasing the generalization of the model. This is defined as:
  (4) $$f_{dropout}\left(x\right)=x\times mask$$
  where mask is a binary tensor that has the same shape as *x*, and its values are set to either 0 or 1 with a probability *p*, determined by the dropout rate. *p* is the fraction of neurons that are kept during each iteration.
  The Softmax function is commonly used in the classification layer to convert the outputs of the network into a probability distribution over the possible classes, stated as:
  (5) $$d_{i}=\frac{\exp\left(y_{i}\right)}{\sum_{j=1}^{c}\exp\left(y_{j}\right)}$$
  where $y_{i}$ is the *i*-th element in the input vector and *c* is the number of classes.

In this model, the Adam optimization algorithm is a popular choice for optimizing neural network models. It is an adaptive learning rate optimization algorithm that adjusts the learning rate dynamically during training to help the network converge to a solution more quickly and accurately.

The learning rate is set to 0.0001, which determines the size of the update steps taken in the direction of the gradient during training. This learning rate can be thought of as a hyperparameter that controls the trade-off between convergence speed and accuracy.

Additionally, the use of early stopping is a commonly used technique to prevent overfitting, which occurs when the network memorizes the training data instead of learning to generalize to new data. Early stopping monitors the validation accuracy during training and stops the training process once the validation accuracy stops improving after a certain number of epochs, which helps prevent overfitting by limiting the training process to the most effective number of epochs.

#### 3.2.2. VGG-16 with Multilayer Perceptron

**Figure 4:**
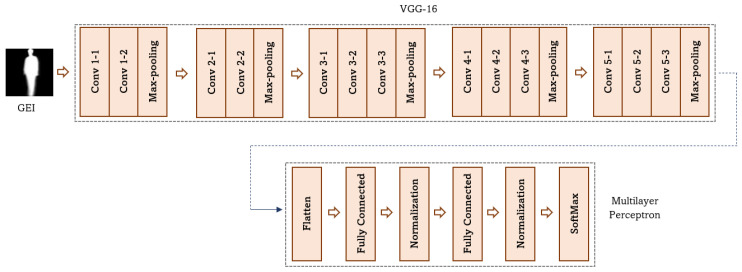
Architecture of the VGG-16 with the Multilayer Perceptron model.

The second pre-trained model employed is VGG-16 model and a Multilayer Perceptron. The VGG-16 model is a CNN model that has been pre-trained on a large dataset and its parameters have already been optimized, making it a popular choice for transfer learning. The architecture of the VGG-16 model is shown in Figure 4.

- VGG-16: The learned features of the pre-trained VGG-16 model [4] are used in this work. The VGG-16 model is similar to the AlexNet model [2], but differs in the number of convolution layers and kernel size. Compared to the AlexNet model, the VGG-16 model has more convolution layers with smaller filter sizes. The model was trained on the ImageNet dataset, which contains 15 million images.
  The VGG-16 model consists of 13 convolution layers, 5 max-pooling layers, and 3 fully connected layers. The convolution layers are divided into five sets, with the first and second sets containing two convolution layers each, and the remaining sets containing three convolution layers each. Max-pooling layers are added after each set of convolution layers.
  A unique aspect of the VGG-16 model is the use of a filter size of 3 × 3 with stride 1 and padding 1 across the entire network. The use of smaller filters helps reduce the number of parameters and avoid overfitting. The original size of the feature maps is reduced by half due to the application of max-pooling over a 2 × 2 pixel window with stride 2.
  The convolution layers in the VGG-16 model use the rectified linear unit (ReLU) activation function to mitigate the vanishing gradient problem. The VGG-16 model was selected due to its computational efficiency, as it has a smaller number of parameters than other models.
- Multilayer Perceptron: The output of the VGG-16 model is vectorized and fed into the MLP. This MLP has a similar architecture to the DenseNet-201 model and consists of two fully connected layers, two batch normalization layers, and a classifier layer. The MLP is used to identify the connection between the encoded features and the class. The MLP is trained to make predictions about the class of an image based on the features extracted by the VGG-16 model. The Adam optimizer with a learning rate of 0.0001 is used to improve network convergence.

#### 3.2.3. Vision Transformer

**Figure 5:**
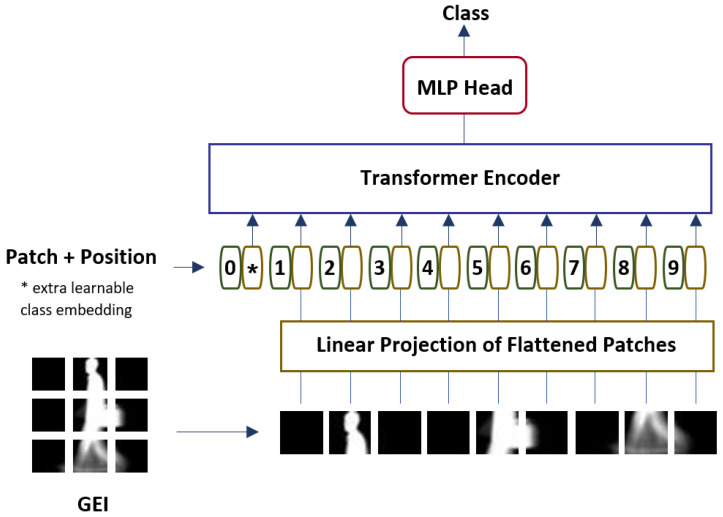
Architecture of the Vision Transformer model.

The third model utilized in this study is the pre-trained Vision Transformer (ViT) model [38], which was pre-trained on both ImageNet and Imagenet-21k datasets. Figure 5 illustrates the architecture of the ViT model. The ViT architecture is composed of the following components:

- Embedding layer: The input image is first divided into a set of non-overlapping patches, and then each patch is transformed into a vector representation. The vector representation of each patch is then fed into the embedding layer to learn the feature representations of the input. The learned representations are then used as the input to the Transformer encoder.
- Transformer encoder: The Transformer encoder consists of multiple layers, each of which is composed of two sub-layers: the multi-head self-attention (MSA) layer and the fully connected feed-forward multilayer perceptron (MLP) layer. The inputs to the encoder are the vector representations of the patches, denoted as $z_{\ell -1}$, where ℓ=1,…,L is the layer index, and *L* is the total number of encoder layers.
  The encoder layer is described as:
  (6) $$z\ell^{\prime }=\mathrm{MLP}\left(\mathrm{MSA}\left(\mathrm{LN}\left(z_{\ell -1}\right)\right)\right)+z_{\ell -1},\;\;\ell =1\ldots L$$
  where LN indicates the layer normalization, MSA denotes the multi-head self-attention layer, and MLP represents the fully connected feed-forward multilayer perceptron.
  Layer normalization is a normalization technique that is applied to the input to the layer in order to improve the performance of the network. It normalizes the values of the input across the feature dimension, which helps to alleviate the problem of vanishing gradients during backpropagation.
  The MSA layer applies self-attention mechanism on the normalized input, which helps the network to better attend to different parts of the input and capture the dependencies between them. The result of the MSA layer is then added to the input to form the final output of the layer. This is done using the skip connections that transfer the information from one layer to another without being affected by the non-linear activation functions in the network. This helps to maintain the gradients during backpropagation and avoid the vanishing gradients problem.
  The final output of the MSA layer is then passed through the MLP, which is a fully connected feed-forward multilayer perceptron. The MLP contains multiple fully connected layers, where Gaussian Error Linear Unit (GeLU) activation function is utilized. GeLU function acts as a regularizer and activation function, where the inputs are multiplied by a value from 0 to 1. The GeLU function’s ability to approximate complex functions are greater than ReLU functions. Other than that, GeLU functions are able to alleviate vanishing gradients problems.
  The final output of the MLP is then added to the input to the MLP, i.e., the output from the MSA layer. The result is the final output of the encoder layer. This operation is also known as residual connection and helps to alleviate the vanishing gradients problem during backpropagation.
  The whole process is repeated for all the *L* encoder layers in the Transformer encoder, and each layer learns to refine the features in the input and output a more comprehensive representation of the input.
- MLP head: The image representation r is generated by applying layer normalization to the output of the last encoder layer $z_{L}^{0}$, which is acquired by:
  (7) $$r=\mathrm{LN}\left(z_{L}^{0}\right)$$
  Subsequently, the image representation r is passed to an MLP head to classify the subjects. The MLP head is a single hidden layer where the sigmoid function is used as the activation function.

#### 3.2.4. Fusion of Prediction

The fusion of predictions in the classification stage is performed using an averaging ensemble technique. The goal of the ensemble is to aggregate the class scores from each model to produce a final decision for a given test sample.

Given a test sample *w*, each model in the ensemble produces a class score $d_{j,m}$ for each class *j* (where j=1,⋯,c and *c* is the number of classes) and the *m*-th ensemble member (where m=1,⋯,M and *M* is the number of ensemble members). These class scores are in the range [0,1], where a higher value indicates a higher probability that the sample belongs to that class.

The final ensemble decision $h_{final}\left(w\right)$ is the class that receives the largest probability. This is computed using the following equation:

(8) $$h_{final}\left(w\right)=\underset{j}{arg max}\mu_{j}\left(w\right)$$

where $\mu_{j}\left(w\right)$ is the final class probability for class *j* and is computed as the average of the class scores from all the ensemble members:

(9) $$\mu_{j}\left(w\right)=\frac{1}{M}\sum_{m=1}^{M}d_{j,m}\left(w\right)$$

The use of an averaging ensemble technique reduces the variance of the individual models, making the estimator more robust and less prone to overfitting. This is because the risk of inaccurate prediction from one model can be diminished by the predictions from other models, which improves the overall performance.

## 4. Experiments and Discussions

In this section, the datasets used for performance evaluation are described, along with the comparison of the results using the existing methods.

### 4.1. Datasets

Three datasets were employed in the evaluation: CASIA-B dataset, OU-ISIR dataset D, and OU-LP dataset.

The CASIA-B dataset [39] contains multi-view gait data that was captured from 124 subjects. The sequences were recorded from 11 different views (0°, 18°, …, 180°) and under 3 different walking conditions (normal walk, walking with a bag, and walking with a coat).

The OU-ISIR dataset D [40] consists of 185 subjects and 370 gait sequences that were recorded from the lateral view. The sequences were divided into 2 groups based on Normalized AutoCorrelation (NAC), with 100 subjects in each group. The DB${}_{high}$ subset has high NAC (small fluctuation), while the DB${}_{low}$ subset has low NAC (large fluctuation).

The OU-LP dataset [41] contains 4016 subjects (1 to 94 years old) and 2 subsets, sequence A and sequence B. Two sequences per subject were recorded in sequence A, and one sequence per subject in sequence B. The sequences were recorded under different observation angles (55°, 65°, 75° and 85°). In this work, only sequence A with 3916 subjects was used.

### 4.2. Selection of Pre-Trained Models

To determine the most suitable pre-trained models for ensemble members, a series of preliminary experiments were performed on the CASIA-B dataset, which is relatively small in size. Pre-trained models, including ResNet-50, EfficientNetB7, DenseNet-201, VGG-16, and ViT, were tested without MLP. Transfer learning was performed on these pre-trained models by using weights from the ImageNet dataset and fine-tuning them on the CASIA-B dataset. A maximum of 300 epochs were run, with early stopping of validation accuracy as the monitoring metric and patience set to 15 epochs. The image size of 64 × 64, batch size 32, and learning rate 0.0001 were used.

The experimental results in Table 1 revealed that DenseNet-201, VGG-16, and ViT achieved the highest accuracies among the pre-trained models, with accuracies of 99.27%, 99.41%, and 99.27%, respectively. The high accuracy of DenseNet-201 can be attributed to its dense connectivity pattern, which allows for better information flow between layers, and its efficient use of memory. VGG-16 achieved high accuracy due to its deep network architecture with multiple convolutional layers, which enables the extraction of complex features from images. The success of ViT can be attributed to its self-attention mechanism, which allows it to attend to different parts of the input image at different scales, without relying on hand-crafted features. This attention mechanism enables ViT to learn more effective representations of the input data, resulting in improved accuracy and generalization.

### 4.3. Ablation Study

An ablation study was conducted to investigate the performance of different ensemble combinations of pre-trained models on the gait recognition datasets, including CASIA-B, OU-ISIR DB${}_{high}$, OU-ISIR DB${}_{low}$, and OU-LP. The study compared the performance of individual models, as well as ensemble models with varying numbers of members. The experimental results of the ablation study are presented in Table 2.

It is observed that the performance of all methods was low when using the CASIA-B dataset due to noise and incomplete silhouettes. However, as the number of ensemble members increased, the accuracy improved significantly. The DenseNet201-MLP + VGG16-MLP + ViT ensemble method achieved the highest accuracy of 100% due to the attention mechanism present in the ViT model. This mechanism dynamically alters the receptive field according to the noise and incomplete silhouettes, resulting in improved accuracy.

For the OU-ISIR DB${}_{high}$ and OU-ISIR DB${}_{low}$ datasets, all methods, including the ensembles, performed promisingly. As the number of ensemble members increased, the accuracy improved further. This is likely due to the integration of pre-trained models and multi-layer perceptron, which are well known for their ability to learn deep features.

Finally, both the DenseNet201-MLP + VGG16-MLP and DenseNet201-MLP + VGG16-MLP + ViT ensemble methods achieved a high accuracy of 99.72% using the OU-LP dataset. These results indicate that having more ensemble members can lead to improved performance in gait recognition tasks.

### 4.4. Comparison with Existing Methods

The proposed multi-model gait recognition system is compared with several existing methods to evaluate its performance. The datasets used for the evaluation are divided into 80% for training, 10% for validation, and 10% for testing.

Table 3 exhibits the performance of the proposed ensemble method, combining DenseNet201-MLP, VGG16-MLP, and ViT on the CASIA-B dataset. The method achieves a state-of-the-art accuracy of 100%, surpassing all existing methods. The improved performance of the proposed method can be attributed to the incorporation of pre-trained models, which are fine-tuned on the gait dataset, enabling it to extract higher-level features effectively. Additionally, the usage of a multi-layer perceptron (MLP) helps to handle the noisy and incomplete silhouettes in the dataset by modeling complex, nonlinear relationships between the extracted features and the target output.

The performance evaluation using OU-ISIR dataset D are shown in Table 4. The proposed ensemble method achieves a remarkable accuracy of 100% on both the DB${}_{high}$ and DB${}_{low}$ subsets, outperforming most of the existing methods. The superior performance of the proposed method can be attributed to the ViT’s unique capability to adjust its receptive field dynamically according to the noise in the input images. The self-attention mechanism of the ViT model enables it to selectively attend to the informative regions of the input image, providing a dynamic and adaptive receptive field that can handle slight variations and occlusions in the walking shapes. Moreover, the ensemble method leverages the strengths of the pre-trained DenseNet201-MLP and VGG16-MLP models in extracting hierarchical features, leading to more robust and accurate predictions.

Table 5 displays the accuracy comparison of the proposed ensemble method with the existing methods on the OU-LP dataset. The proposed method achieved a remarkable accuracy of 99.72%, outperforming all existing methods. The superior performance of the proposed method can be attributed to the multi-head attention mechanism of the ViT model, which allows it to attend to different parts of the image, capturing diverse and relevant information. The resulting features are concatenated with strong skip connections, enhancing the generalization and scalability of the model. Additionally, the proposed ensemble method uses the averaging ensemble technique, which combines the prediction scores of individual models to improve the overall performance. This technique allows the strengths of individual models to complement each other, leading to better predictions.

## 5. Conclusions

The paper presents a multi-model gait recognition system that leverages the strengths of three different deep learning models: DenseNet-201, VGG-16, and Vision Transformer. The input to the system is a gait energy image that is computed by windowing the gait frames over a gait cycle. Each of the three models then takes this gait energy image as input and produces prediction scores by learning the gait features. Finally, the prediction scores from all three models are combined and averaged to generate the final prediction. The proposed system is robust against incomplete and noisy silhouettes, as it incorporates the self-attention mechanism from the Transformer model. The combination of CNN models and the Transformer model improves the generalization capability for gait recognition. The implementation also includes techniques such as early stopping, skip connections, and layer normalization to prevent overfitting and reduce the computation cost. Additionally, the ensemble technique reduces the variance among the models, thereby increasing the overall system efficiency.

## Figures and Tables

**Table 1:** Experimental results of the pre-trained models on the CASIA-B dataset.

Models	Accuracy (%)	Time (s)
ResNet-50	98.46	363.4562
EfficientNetB7	97.56	2756.6737
DenseNet-201	99.27	772.2298
VGG-16	99.41	1434.2785
ViT	99.27	2674.7809

**Table 2:** Experimental results of the different ensemble models.

Methods		Accuracy (%)			
		CASIA-B	OU-ISIR DB${}_{\mathrm{high}}$	OU-ISIR DB${}_{\mathrm{low}}$	OU-LP
DenseNet201-MLP		99.71	100	100	99.45
VGG16-MLP		99.85	100	100	98.53
ViT		99.27	100	99.93	95.69
DenseNet201-MLP + VGG16-MLP		99.93	100	100	99.72
DenseNet201-MLP + VGG16-MLP + ViT		100	100	100	99.72

**Table 3:** Comparison results on the CASIA-B dataset.

Methods	Accuracy (%)
GEINet [42]	97.65
Deep CNN [43]	25.68
CNN [44]	98.09
CNN [45]	94.63
Deep CNN [46]	86.17
GCF-CNN [31]	83.53
L-SVM [47]	98.20
Gait-CNN-ViT (Proposed)	100

**Table 4:** Comparison results on the OU-ISIR dataset D.

Methods	Accuracy (%)	
	OU-ISIR DB${}_{\mathrm{high}}$	OU-ISIR DB${}_{\mathrm{low}}$
GEINet [42]	99.93	99.65
Deep CNN [43]	87.70	83.81
CNN [44]	99.65	99.37
CNN [45]	89.99	96.73
Deep CNN [46]	96.18	95.21
Gait-CNN-ViT (proposed)	100	100

**Table 5:** Comparison results on the OU-LP dataset.

Methods	Accuracy (%)
GEINet [42]	90.74
Deep CNN [43]	5.60
CNN [44]	89.17
CNN [45]	48.32
Deep CNN [46]	45.52
DeepGait [27]	98.95
CVGR-EL [48]	97.10
GCF-CNN [31]	86.04
Gait-CNN-ViT (proposed)	99.72

## Data Availability

Not applicable.
